# The minimal amount of starting DNA for Agilent’s hybrid capture-based targeted massively parallel sequencing

**DOI:** 10.1038/srep26732

**Published:** 2016-05-25

**Authors:** Jongsuk Chung, Dae-Soon Son, Hyo-Jeong Jeon, Kyoung-Mee Kim, Gahee Park, Gyu Ha Ryu, Woong-Yang Park, Donghyun Park

**Affiliations:** 1 Samsung Biomedical Research Institute, Samsung Advanced Institute of Technology, Samsung Electronics Co. Ltd., Seoul 06351, Korea; 2 Samsung Genome Institute, Samsung Medical Center, Seoul 06351, Korea; 3 Department of Molecular Cell Biology, Sungkyunkwan University School of Medicine, Suwon 16419, Korea; 4 Department of Pathology and Translational Genomics, Sungkyunkwan University School of Medicine, Suwon 16419, Korea; 5 Office of Research and Development Strategy & Planning, Samsung Medical Center, Seoul 06351, Korea

## Abstract

Targeted capture massively parallel sequencing is increasingly being used in clinical settings, and as costs continue to decline, use of this technology may become routine in health care. However, a limited amount of tissue has often been a challenge in meeting quality requirements. To offer a practical guideline for the minimum amount of input DNA for targeted sequencing, we optimized and evaluated the performance of targeted sequencing depending on the input DNA amount. First, using various amounts of input DNA, we compared commercially available library construction kits and selected Agilent’s SureSelect-XT and KAPA Biosystems’ Hyper Prep kits as the kits most compatible with targeted deep sequencing using Agilent’s SureSelect custom capture. Then, we optimized the adapter ligation conditions of the Hyper Prep kit to improve library construction efficiency and adapted multiplexed hybrid selection to reduce the cost of sequencing. In this study, we systematically evaluated the performance of the optimized protocol depending on the amount of input DNA, ranging from 6.25 to 200 ng, suggesting the minimal input DNA amounts based on coverage depths required for specific applications.

Knowledge of somatic genomic alterations in tumors that informs therapeutic options has been facilitating individualized approaches to cancer treatment^1^,^2^. Comprehensive characterization of cancer genome was enabled by the advent of massively parallel sequencing, which is transforming the cancer genomics landscape^3^. Even with reductions in the cost-per-base of DNA sequencing, routine sequencing of large numbers of whole eukaryotic genomes is not always feasible for several reasons. As an alternative approach, enrichment of particular genomic regions prior to sequencing was developed to reduce sequencing costs and simplify analysis^4^,^5^,^6^,^7^,^8^. While targeted sequencing is now widespread, targeted sequencing techniques to enrich for cancer-related genomic regions have been successfully employed for clinical implementation of systematic genomic profiling^9^.

At the same time, however, obstacles posed by clinical tissue samples have been repeatedly noted^10^,^11^. The challenges include that clinical tissue samples are often minute in quantity as needle biopsies become common^12^,^13^. Since clinical samples are nonrenewable, specimen yield is often a limiting factor determining the types of techniques that can be employed. Initially, targeted sequencing was developed to use large amounts of input DNA generated from blood samples. Improvements in targeted deep sequencing or whole-exome sequencing have been attempted to lower the amount of input DNA required, but most protocols still required 200 ng or more of genomic DNA^14^,^15^. One of the reasons why relatively large amounts of DNA are required for targeted sequencing is the need to detect somatic mutations present at low mutant allele frequency (MAF). Somatic mutations frequently occur in only a subset of the sequenced cells owing to tumor heterogeneity. Detection of such mutations, particularly at a stage before they become dominant in the population, could be essential to optimizing therapy^16^. Since high levels of normal cell contamination is another common phenomenon, significant dilution of the mutant alleles of interest is not rare at all. Thus, a useful clinical assay for cancer genome profiling has to perform well with a limited amount of tissue to be sensitive enough to detect low levels of mutations and accurate enough to minimize false-positive reports. In addition, a single, tissue-sparing test that maximizes information retrieval is preferred.

Although small amounts of input DNA, as little as 10–70 ng, have been used successfully to analyze germline variants in a homozygous or heterozygous state^17^,^18^,^19^, the library complexity required to detect variants present at 100% or 50% allele frequency is much lower than somatic variants with allele frequencies as low as 5%. While the technology has been rapidly evolving, the effect of the input DNA amount on performance of targeted sequencing has not been systematically evaluated, especially for the detection of low allele frequency variants.

Here, we systematically evaluated the performance of our targeted sequencing protocol with various amounts of input DNA, with respect to library complexity, coverage efficiency, and variant discovery. Beforehand, we optimized our targeted sequencing protocol based on Agilent’s SureSelect custom capture to meet the requirements for clinical cancer genome profiling. For this purpose, we first compared the performance of five commercially available kits including Agilent’s SureSelect XT and QXT, KAPA Biosystems’ Hyper Prep, Rubicon’s ThruPLEX DNA-seq, and NEB’s NEBNext Ultra DNA Library Prep Kits, because their performance for targeted deep sequencing has not yet been compared, despite vendors’ claims of superior library yields and limited amplification bias. Next, we optimized the adapter ligation conditions to increase library construction efficiency. We also adapted and evaluated multiplexed pre-enrichment pooling with a single hybridization. By employing our optimized protocol, we evaluated the effect of DNA input amount on analytical performance and suggested a minimal input DNA amount for targeted sequencing.

## Results

### Comparison of five library construction kits

**Figure 1:**
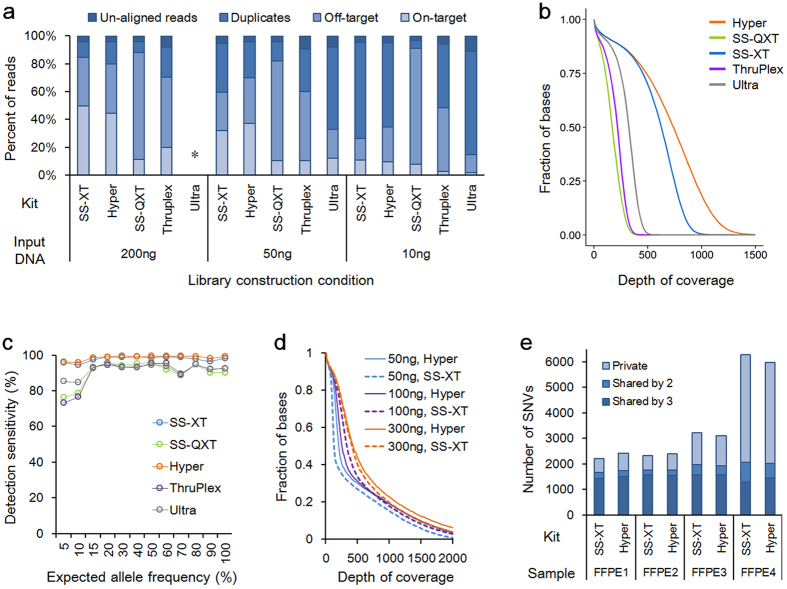
Comparison of kits using HapMap cell line DNA (**a–c**) and FFPE samples (**d,e**). (**a**) Stacked bar plot showing subgroups of filtered reads and reads left after filtering (i.e., on-target) for five commercial kits using 200 ng, 50 ng, and 10 ng of input DNA. The NEBNext Ultra kit failed to construct an acceptable library using 200 ng of input DNA (indicated by an asterisk). (**b**) Coverage efficiency comparison using 50 ng of input DNA (n = 2). Coverage efficiency is visualized as the percent of the total targeted bases covered at particular depths. Data in (**a**,**b**) were averaged from two sets of libraries from two HapMap pools (10 cell lines each). (**c**) Base substitution detection sensitivity of libraries using 50 ng of input DNA was plotted against expected allele frequencies of base substitution alterations (x-axis). (**d**) Coverage efficiency comparison using an FFPE sample (FFPE1 in **e**). (**e**) Comparison of base substitution concordance among libraries from the same sample. For each of four samples, three libraries were generated using 300 ng, 100 ng, or 50 ng of input DNA. Depending on the number of libraries detecting a variant, variants were classified into three groups; private to a library, shared by two libraries, or found in three libraries.

Utilizing Agilent’s SureSelect capture technology, whose performance has been well validated^5^,^20^,^21^,^22^, we compared the performance of library construction kits that are commercially available. Briefly, purified genomic DNA from two pools of normal cell lines were used to construct libraries using commercial kits and hybridization-based capture of 6839 exons from 381 cancer-related genes and introns from 23 genes. Using the Illumina HiSeq2500 platform, hybrid-capture-selected libraries were sequenced to high depth, targeting 50 million total reads for each library. To evaluate how each kit performed with a limited amount of input DNA, we used different input amounts, 200 ng, 50 ng, and 10 ng, for each library construction kit. The libraries for the kits were prepared according to each supplier’s recommended protocol as described in Materials and Methods. For target enrichment, an identical pool of RNA baits targeting ~2.2 Mb of human genome was used to perform solution hybridization of all these libraries as described in Methods. On average, 49.5 million reads of each sample were generated, varying between 38.2 and 64.2 million reads. The read alignment rate was 94%, on average, varying between 89% and 97.2% (Supplementary Table S1). After read alignment, mapped reads of each sample were filtered for duplicates, improper pairs, and off-target reads using PICARD and SAMtools. At all three input amounts, SureSelect-XT and Hyper kits showed a higher pass-filter rate than the other kits (Fig. 1a).

We compared coverage features for the target region, such as uniformity and coverage depth. To adjust the data size of each sample to a comparable number of total reads, *in silico* down-sampling (the random selection of a subset of reads) was carried out. Total reads of each sample were down-sampled to 38.2 million, the smallest data size among all data sets. At this level of total read count, SureSelect-XT and Hyper kits also showed higher mean coverage than the other kits (Fig. 1b, Supplementary Fig. S1, and Supplementary Table S1). In the down-sampled data set generated by using 50 ng of DNA, SureSelect-XT and Hyper kits showed 654× and 798× coverage, on average.

To validate the performance of base substitution detection, we employed a method previously reported and used a pool of 10 normal cell line HapMap samples from ATCC^9^. According to the SureSelect-XT protocol, 200 ng of initial genomic DNA from each normal cell line was indexed separately and sequenced to obtain base substitutions. Based on the abundance and population distribution of verified germline base substitution variation in each cell line, the pool of 10 cell lines was expected to have 4840 base substitutions that spanned a broad range of MAF (5–100%) across the targeted regions (Supplementary Fig. S1 and Supplementary Table S2). As detection sensitivity was tightly associated with depth of coverage, especially for mutations with low allele frequency, SureSelect-XT and Hyper kits displaying relatively high mean coverage also showed high detection sensitivity: >95% of base substitutions expected to be present at MAF ≥ 5% using 200 ng and 50 ng of input DNA (Fig. 1c and Supplementary Fig. S1).

While detection of small DNA subpopulations requires deep sequencing a sufficient number of molecules, a practical limit of detection is also imposed by errors introduced during sample preparation and sequencing^23^,^24^. To measure the unexpected base substitution error rate, background alleles common to all individual cell line constituents were listed and examined in the test specimens derived from the pool. Mean background error rates were similar among samples (0.015% ± 0.004). However, the distributions of the background error rate from the SureSelect-XT and Hyper kit were more left-shifted than the other three methods due to differences in the mean depth of coverage, which implies less false positive calls in the SureSelect-XT and Hyper kit (Supplementary Fig. S2).

Based on these results, we selected the SureSelect-XT and Hyper kit for further performance evaluation using formalin-fixed, paraffin-embeded (FFPE) samples. Four FFPE specimens with a variable degree of DNA quality were chosen (Supplementary Fig. S3). For the comparison, 300 ng, 100 ng, and 50 ng of input DNA extracted from each of four FFPE blocks were used for library construction. Data from each sample were down-sampled to 39.3 million reads, adjusting data size of every sample to the smallest data set. While mean depth of coverage correlated with the amount and quality of input DNA, the SureSelect-XT and Hyper kits showed similar mean depth of coverage overall (Fig. 1d and Supplementary Table S3). Base substitution detection performance was indirectly accessed by concordance among the three libraries from the same sample. The better the sensitivity and specificity that is achieved in single nucleotide variation (SNV) detection, the more SNVs that were shared by the three libraries from each sample. While the number of SNVs unique to a single library was increased in samples with poor DNA quality, as expected, the difference between SureSelect-XT and Hyper kits was not significant (Fig. 1e). Taken together, the SureSelect-XT and Hyper kits performed better under specific conditions, such as targeted deep sequencing employing Agilent’s SureSelect custom capture, although we did not generalize their performance to other applications.

### Optimization of library construction

**Figure 2:**
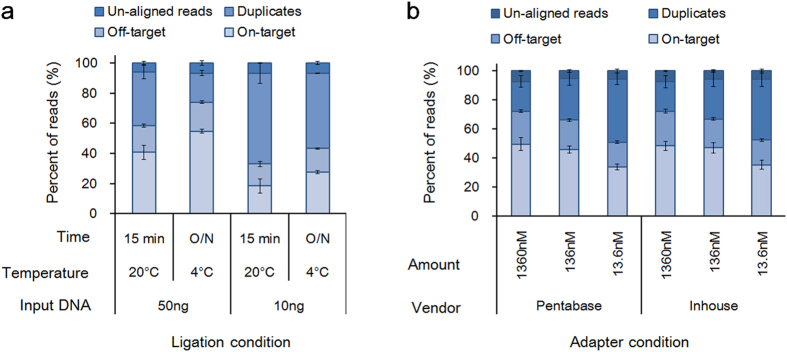
Optimization of adapter ligation. Stacked bar plot showing subgroups of filtered reads and reads left after filtering (i.e., on-target) for each condition. (**a**) Effect of ligation temperature. Ligation using 50 ng or 10 ng of input DNA was performed in duplicate either at 25 °C for 15 min or 4 °C overnight. (**b**) Effect of adapter concentration. Values are expressed as mean ± s.e.m. (n = 3).

Protocols for library construction were optimized in a step-wise manner with the goal of maximizing adapter ligation efficiency, consequently reducing the number of PCR cycles needed following adapter ligation. First, commercially available kits were screened to find the best kit for our purpose, as described above. Next, further optimization of adapter ligation conditions was performed using the selected kit. We optimized the i) temperature and duration of ligation reaction and ii) molar ratio of adapter to template. For the purpose, KAPA Biosystems’ Hyper Prep kit was chosen owing to its streamlined workflow, resulting in a shorter hands-on time. Initially, we screened conditions for ligation temperatures ranging from 16 °C to 25 °C for 15 min or 60 min. Compared with the standard conditions that the manufacturer recommended (i.e., 20 °C for 15 min), none of these conditions resulted in significant improvements (data not shown). When ligation at 4 °C overnight was compared with ligation in the standard conditions, the duplication rate was significantly decreased (Fig. 2a). Consequently, reads after filtering improperly mapped reads, duplicates, improper pairs, and off-target reads were increased from 40% to 55% or from 18% to 28% using 50 ng or10 ng of input DNA, respectively.

Next, we replaced the SureSelect-XT adapter with an indexed adapter for pooling hybrid selection. To determine the optimal adapter concentration, we tested different adapter concentrations, ranging from 13.6 nM to 1.36 μM, which corresponded to adapter:insert molar ratios from 300:1 to 30000:1 using 50 ng genomic DNA as an input. For the test, ligation was carried out at 4 °C overnight. Adapter oligonucleotides from two independent sources were used to rule out a vendor effect. A Y-shaped adapter was used to preserve the directionality of each single-stranded DNA (ssDNA) and to allow amplified DNA on the flow cell to be unidirectional^25^. One strand of the adapters contained barcode sequences, which allow multiplexing of multiple libraries into one hybridization reaction. A Pentabase adapter was chosen among the ones commercially available, because the adapter concentration was specified. Pre-indexed adapter oligonucleotides were also synthesized by Integrated Device Technology (IDT), Inc. and tested in parallel. After sequencing the libraries for the conditions, we down-sampled the data to a 1233× raw read depth, equalizing the average data sizes of Fig. 2a,b. The results were very similar between the adapter oligonucleotides from the two sources. Although the difference between using 1.36 μM and 13.6 nM was minute in the libraries prepared from 50 ng of input DNA, the highest adapter concentration produced the best overall results, suggesting that excess adapter concentrations increased the efficiency of ligation (Fig. 2b). Whereas a high adapter concentration might have resulted in significantly more adapter dimers, the purification step performed after ligation seemed to be sufficient to clean up adapter dimers in the libraries. Thus, the optimized protocol using the highest adapter concentration and ligation at 4 °C overnight was evaluated later for performance, depending on the amount of input DNA.

### Pooled hybridization capture

**Figure 3:**
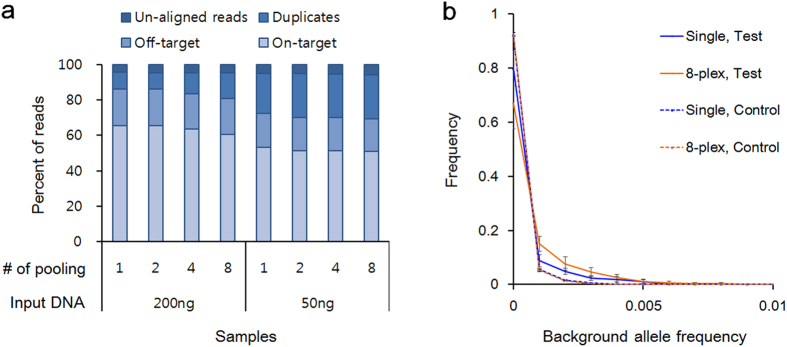
Pooled hybridization capture. (**a**) Stacked bar plot showing subgroups of filtered reads and reads left after filtering (i.e., on-target), depending on the input DNA amount and the number of samples in a single hybrid selection reaction. (**b**) Analysis of cross-contamination. Cell line-specific base substitutions absent in a sample of interest and present in the other samples multiplexed in the same hybrid selection reaction were selected as a ‘Test’ group, whereas alleles absent in all samples were chosen as a ‘Control’ group. Background rates in both groups were assessed for either single- or eight-plex hybrid selection. The x-axis denotes the frequency of background alleles, and the y-axis denotes the fraction of alleles with the designated background rate on the x-axis. Details are given in Materials and Methods.

We adapted our protocol for multiplex hybrid selection (pooling multiple libraries prior to capture hybridization) since it would reduce the cost of the target enrichment step and provide higher throughput. Indexed libraries were constructed, and hybrid selection was performed in multiplex. First, we examined if hybrid selection performed in multiplex formats compromised data quality. For the test, libraries were generated from 200 ng and 50 ng of input DNA from normal cell lines (Supplementary Table S4) and subjected to hybrid selection. When libraries in one hybridization reaction were increased up to 8, all sequencing metrics, such as duplication rate, on-target rate, and target read depth, were comparable with single-plex hybridization results (Fig. 3a).

Second, we scrutinized if there was any cross-contamination in multiplexed samples, although libraries were indexed prior to capture. Besides extremely common low-level DNA contamination in laboratories, index swapping on the sequencer in the absence of a physical sample contamination—a phenomenon whose cause is not well understood—can result in a few reads from one patient being ascribed to another patient. Multiplexed hybrid selection might also lead to additional cross-contamination, which was evaluated by analyzing cell line-specific single nucleotide polymorphisms (SNPs) across samples, as described in Materials and Methods. SNPs absent in a sample of interest but present in the other multiplexed samples in a capture hybridization reaction were regarded as a test group whose background rate was potentially influenced by cross-contamination. The background rate in the test group was examined if their allele frequencies (i.e., background rate) were influenced by multiplexed hybrid selection. The mean background rate in the test group was slightly increased to 0.077% in an eight-plex hybridization from 0.052% in single-plex hybridizations. As a control group, the error rate of background alleles common to all eight samples were compared between the single- and eight-plexed hybrid selection, because background rates in this group were not influenced by cross-contamination. As expected, the distributions of error rates from the single- and eight-plexed hybrid selection were superimposed on each other in the control groups (Fig. 3b). In the control groups, we found mean background rates of 0.012% or 0.011% in single-plex or eight-plex hybridization, respectively. Thus, without a change in the intrinsic error rate, the increase in cross-contamination from pooled hybrid selection was estimated at 0.025%, which would not be a problem for most applications.

### Performance of the optimized protocol

**Figure 4:**
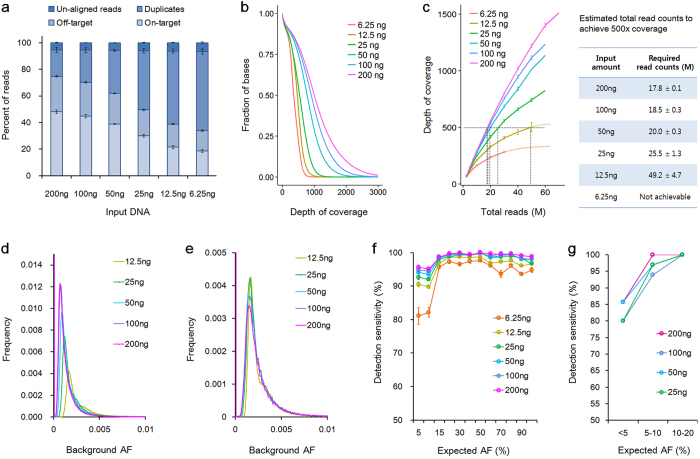
Performance of the optimized targeted deep sequencing protocol depending on input DNA amount. (**a**) Stacked bar plot showing subgroups of filtered reads and reads left after filtering (i.e., on-target), depending on the input DNA amount. (**b**) Coverage efficiency depending on the input DNA amount. Coverage efficiency was visualized as the percent of the total targeted bases covered at particular depths. Data in A and B was averaged from two sets of libraries from two HapMap pools (10 cell lines each). (**c**) Mean depth of coverage after filtering was plotted against total read depth. (**d,e**) Background error rate was analyzed from data sets down-sampled to a total of 40 M read counts (**d**) or mean depth of coverage of 500× (**e**). The x-axis denotes the frequency of background alleles, and the y-axis denotes the fraction of alleles with the designated background rate on the x-axis. (**f**) Base substitution detection sensitivity of libraries was plotted against expected allele frequencies of base substitution alterations (x-axis). (**g**) Indel detection sensitivity of libraries was plotted against expected allele frequencies of base substitution alterations (x-axis). Details are described in Materials and Methods. Statistical variation for (**a,c,f**) is shown as mean ± s.e.m (n = 3 or 4).

After the optimization of library construction and adoption of multiplexed hybrid selection, the targeted deep sequencing protocol was evaluated for its performance depending on input DNA amounts of 200, 100, 50, 25, 12.5, and 6.25 ng. On average, 51.3 million reads were generated from each sample, varying between 25.5 and 68.9 million reads. The read alignment rate was 94%, on average, varying between 92.3% and 95.1% (Supplementary Table S5). After read alignment, mapped reads from each sample were filtered for duplicates, improper pairs, and off-target reads as described above. Overall, the percentage of pass-filter mapped reads tightly correlated with the amount of input DNA, primarily due to a negative correlation between the duplicate rate and DNA amount (Fig. 4a). As a result, depth of coverage was positively correlated with DNA amount (Fig. 4b). While target regions covered >100× exceeded 90% in all cases, the percentage of targets covered with high depth varied dramatically depending on the input DNA amount. For example, the percentage of targets covered >500× was 80.1% in the sample with 200 ng of input DNA, but dropped to 22.7% with 6.25 ng of DNA. Since data from each sample were down-sampled to 35.7 million reads in order to adjust the data size of every sample to the smallest data set for fair comparison, inferior mean depth of coverage in low input samples indicated their low library complexity. Low complexity DNA sequencing libraries are often problematic, especially in targeted deep sequencing: many sequenced reads will correspond to the same original molecules, and deeper sequencing either provides redundant data that is discarded, or introduces biases in downstream analyses.

Depending on the input DNA amount, the mean depth of coverage of total read counts was further evaluated by an *in silico* down-sampling of data (Fig. 4c). Over a wide range, from 2.5 M up to 68.9 M, of total read counts, coverage depth changes were compared among libraries using different amounts of input DNA. For instance, all samples except 6.25 ng of input achieved a >500× mean depth of coverage, but total read counts required to achieve a 500× mean depth of coverage significantly varied, based on differences in library complexity that were dependent on the amount of input DNA (Fig. 4c). We concluded that 6.25 ng of input DNA was amenable to attaining more than 200× mean depth coverage, but the benefit had to be weighed against the high coverage demands and potential challenges to detect SNV and indels with low MAF.

Next, we examined whether background error rates were influenced by input DNA amounts. Mean background error rates were similar among samples (0.021 ± 0.001%) (Fig. 4d). In addition, when the total read counts were adjusted to achieve equal coverage (500×), the frequency distributions of the background rates were superimposed in all samples (Fig. 4e). However, with a randomly selected 40 million total reads for each sample, the coverage differences also resulted in changes in the distributions of background error rates (Fig. 4d). This result implied that relatively high frequency errors would occur more often in libraries using lower amounts of input DNA, because of lower mean depth of coverage.

To evaluate the detection sensitivity of SNV, a pool of 10 normal cell line HapMap samples was used as described above. The overall substitution detection performance was high, as >96% of base substitutions expected to be present at MAF ≥ 15% were successfully detected in all libraries using amounts of input DNA as low as 6.25 ng. However, detection sensitivity of substitutions at MAF ≤ 10% in samples using 12.5 ng or 6.25 ng was compromised to ~90% or below compared with 92.3–95.4% in the other samples (Fig. 4f). To measure indel detection sensitivity, we used 10 tumor cell lines containing a total of 35 indel alterations in 20 genes to generate three pools of five or 10 cell lines each, thereby creating a test set of 70 indels spanning MAF < 20% and indel lengths (1–36 bp) (Supplementary Table S6). A range of DNA input amounts (between 25 ng and 200 ng) was tested, because 25 ng is the smallest amount that displayed comparable performance in base substitution detection as that with 200 ng (Fig. 4g and Supplementary Table S7). As was the case for base substitutions, 25 ng showed little compromise in indel detection sensitivity: 97% (32/33) of indels at 5% ≤ MAF ≤ 10% were successfully detected, as well as 80% (28/35) of indels at MAF ≤ 5%.

## Discussion

Input DNA amounts required for a specific application vary greatly, as requirement for detection limits, based on allele frequencies, depends on the application. The applications that require detection of low allele frequency mutants are manifold in cancer for several reasons. When mutations are assessed in tissue biopsies, tumor heterogeneity and/or normal cell contamination require sensitive detection of mutants present at low allele frequencies. Since the sensitivity, specificity, accuracy, and precision are tightly related to mean depth of coverage, the practically achievable mean depth of coverage, which depends on amount of input DNA, would offer a foundation for guidelines on the required DNA amount. Thus, we systematically analyzed the relationship between mean depth of coverage and input DNA amount.

We found that the data generated from 25 ng of cell line genomic DNA using the modified protocol was sufficient to achieve >700–800× mean depth of coverage (Fig. 4c). Although the manufacturer recommended 200 ng of starting genomic DNA, decreasing the input DNA from 200 ng to 25 ng by using the modified protocol resulted in a relatively small impact on targeted sequencing quality and variant-calling consistency and maintained high sensitivity for the detection of SNVs and indels at 5% MAF or greater. Further decreasing input DNA, as low as 6.25 ng, we were still able to achieve ~300× mean depth of coverage, displaying an SNV detection sensitivity >96% for SNVs at MAF ≥ 15%. However, the detection sensitivity of SNVs at MAF ≤ 10% was significantly compromised (Fig. 4f). Based on the detection sensitivity of SNVs and indels, our data suggested that input DNA amounts as small as 25 ng should be satisfactory for next-generation sequencing (NGS)-based clinical cancer gene tests.

Previously, Frampton, G.M. *et al*. showed that 250× median coverage was sufficient to detect >99% of base substitutions present at MAF ≥ 10% and 98% of substitutions at 5% ≤ MAF ≤ 10%, which is desirable for NGS-based clinical cancer gene tests^9^. Wagle, N. *et al*. also demonstrated that cancer mutations in FFPE samples were accurately detected by achieving 400-fold mean sequence coverage^26^. Consistently, our unpublished data (manuscript in preparation) via *in-silico* simulation also estimated that 300× mean depth of coverage was required to assure >95% detection sensitivity of 5% SNVs. Therefore, attainable mean depth of coverage using 25 ng of genomic DNA exceeded the coverage requirement for capture-based clinical cancer gene sequencing. Although lowering the input amount below 25 ng for such applications demands further optimization and validation of the technique, input amounts of DNA as low as 6.25 ng are expected to be sufficient for applications targeting a mean depth of <300× such as whole-exome sequencing or targeted sequencing dealing with homogenous samples.

For some applications, detection of rare mutations present even at ~1% or less MAF is desired^27^,^28^. Sensitive methods for detecting tumor mutations may find use in early detection screening, determination of prognosis, monitoring tumor dynamics over time, or detection of minimal residual disease, when rare mutations are evaluated in stool, sputum, plasma, and other bodily fluids^27^,^29^,^30^,^31^. As DNA amounts are frequently limited in these applications to detect mutations in cell-free DNA (cfDNA), it is critical to achieve sufficient read depths from small amounts of DNA. In fact, employing the modified protocol for analyzing cfDNA, we routinely achieved ~10000× mean depth coverage before duplicate removal and 2500~3000× after duplicate removal from 50 ng of cfDNA. Obviously, the minimal input DNA amount varied from less than 10 ng to more than 50 ng for targeted sequencing, depending on the library complexity or mean depth coverage required for a specific application.

Previous studies have indicated that coverage depths in the range of 250–400× were sufficient for capture-based clinical cancer gene sequencing. However, for the same application, we suggested 25 ng of input DNA by which >700–800× coverage depth is achievable. The discrepancy might be a result of target regions with low coverage bias in our panel. Although SureSelect capture platform showed relatively decent library complexity and good uniformity^21^,^22^, a small portion of target regions in our customized panel displayed relatively poor coverage, regardless of the input DNA amount. Consequently, failure to detect variants was more frequent in these regions. When the target regions with low coverage bias were removed, we observed a significant improvement in the sensitivity of variant detection. For instance, when positions showing below 200× mean coverage calculated from 22 samples using 200 ng of input DNA were removed, 6.25 ng of input DNA was sufficient to detect ≥ 99% of base substitutions expected to be present at MAF ≥ 15% (Supplementary Fig. S4). These data were consistent with a previous notion that uniform coverage across all regions of interest is essential for sensitivity in detecting variants. In addition to average depth of coverage as an important metric, equal distribution of coverage across all regions of interest is also critical for ideal targeted sequencing. Coverage non-uniformity might be attributed to coverage-reducing biases of sequencing, notably at GC extremes, palindromes, and inverted repeats, as well as capture efficiency bias^32^,^33^,^34^. Although it is difficult to estimate how much coverage-reducing bias can be attributed to uneven capture efficiency, the optimization of probes displaying poor recovery efficiency, such as increasing the probe density, might improve detection sensitivity of custom-targeted DNA capture sequencing.

We generated sequencing data with various amount of total reads depending on the purposes of the experiments. Consequently, direct comparisons of sequencing metrics among the experiments were not feasible due to the significant data size differences. For a fair comparison, we down sampled the data sets for comparison of library construction kits (Fig. 1a) and performance evaluation of the optimized protocol (Fig. 4a) to a 1233× raw read depth, which were the average size of the data set for condition optimization (Fig. 2). The results showed consistency in our experiments (Supplementary Fig. S5). For instance, the average on-target rates using 50 ng DNA input under the condition of optimized protocol were 48.4% in Fig. 2b and 49.6% in the down-sampled data from Fig. 4a.

The minimal requirement for the input DNA amount varies greatly, depending not only on the performance requirements for specific applications but also on the efficiency of the method employed. To improve the efficiency of the standard protocol, we screened commercial kits, chose the KAPA Hyper kit, optimized adapter ligation efficiency, and adopted multiplexed target capture. Obviously, as methods with different efficiencies need variable amounts of input DNA, the performance of targeted sequencing that is dependent on input DNA amounts described here is based on the optimized protocol.

In conclusion, this study provides a comprehensive evaluation of the impact of DNA input amount on analytical performance using the optimized targeted sequencing protocol. Potential applications that might benefit from this evaluation include a variety of cancer genome profiling assays, especially when sample requirements specified by manufacturer’s standard protocol cannot be readily met.

## Materials and Methods

### Patient samples

Tumor specimens were collected from patients with lung cancer undergoing surgical resection. All tumor specimens used in the study were paraffin embedded tumor tissues. The study was approved by the institutional review board at Samsung Medical Center and all the methods were carried out in accordance with the approved guidelines. Written informed consent was obtained from all subjects.

### DNA extraction

For the evaluation of analytical performance, purified DNA from 20 normal HapMap cell lines (Supplementary Table S1) were purchased from the Coriell Institute (http://ccr.coriell.org/). For indel validation, 10 immortalized tumor cell lines (Supplementary Table S2) were purchased from ATCC (American Type Culture Collection, http://www.atcc.org). Genomic DNA was extracted from cell lines using QIAamp DNA Mini Kits (Qiagen, Valencia, CA, USA). In the case of FFPE clinical samples, DNA was extracted from 40 μm of unstained FFPE sections, typically 4 × 10 μm sections, using a Promega Maxwell 16 CSC DNA FFPE kit with an automation instrument (Promega, Fitchburg, WI, USA). DNA concentration and purity were assessed by a Nanodrop 8000 UV-Vis spectrometer (Thermo Scientific, Waltham, MA, USA) and a Picogreen fluorescence assay using a Qubit 2.0 Fluorometer (Life Technologies, Grand Island, NY, USA). The fragment size distribution, indicating the degree of DNA degradation, was measured using a 2200 TapeStation Instrument (Agilent Technologies, Santa Clara, CA, USA) and real-time PCR Mx3005p (Agilent Technologies, Santa Clara, CA, USA) according to the manufacturer’s manual.

### Library preparation

A total of five library preparation kits were tested: SureSelect-XT (Agilent Technologies, Santa Clara, CA, USA), SureSelect-QXT (Agilent Technologies, Santa Clara, CA, USA), KAPA Hyper (Kapa Biosystems Inc., Massachusetts, USA), NEBNext Ultra (New England Biolabs, Inc., Massachusetts, USA), and ThruPLEX kit(Rubicon Genomics, Miami, USA). To make the library using all kits except for SureSelect-QXT, genomic DNA was fragmented to 150–200 bp by sonication using a Covaris S2 (7 min, 0.5% duty, intensity = 0.1, 50 cycles/burst; Covaris Inc.) followed by purification using a 1.8× volume of AMPure XP Beads (Beckman Coulter, Indiana, USA). The SureSelect-QXT protocol utilized an enzymatic fragment process instead of sonication. After the fragmentation process, end-repair, A-tailing, adapter ligation, and PCR reactions before target enrichment was performed, following the manufacturer’s recommended protocols. After each step, the purification step was performed with AMPure beads to remove short fragments such as adapter dimers. Different adapters were used for comparison of kits according to each manufacturer’s protocol. Pre-indexed adapters were utilized for multiplexing hybrid selection.

### Target enrichment

Agilent SureDesign was used to design unique RNA baits, which targeted ~2.2 Mb of human genome, including 6839 exons from 381 cancer-related genes and introns from 23 genes frequently rearranged in solid tumors. For comparison of library construction kits and performance evaluation of the optimized protocol, solution hybridization was accomplished using the RNA baits. For the optimization of ligation conditions and the cross-contamination estimation, a subset of the RNA baits targeting ~0.5 Mb regions were used. After pre-amplification of libraries, double-stranded DNA concentrations were measured by Qubit Fluorometer (Life Technologies, Grand Island, NY, USA) and fragment size distributions were assessed by a 2200 TapeStation Instrument (Agilent Technologies, Santa Clara, CA, USA). The libraries were adjusted to a total of 750 ng of DNA for each hybrid selection reaction as recommended by the SureSelect bait hybridization protocol. For comparison of commercial kits, target enrichment was performed according to each manufacturer’s recommended protocol. While SureSelect’s blocking oligonucleotide was used for SureSelect-XT, SureSelect-QXT, and KAPA Hyper kits, IDT xGen blocking oligonucleotide (IDT, CA, USA) was used for NEBNext Ultra and ThruPLEX kits. This was because the latter included sample-specific index sequences in their adapter at the hybrid selection step, but the former did not.

### Sequencing

Based on DNA concentration and average fragment size, libraries were normalized to an equal concentration, 2 nM, and pooled by equal volume. After denaturing libraries using 0.2 N NaOH, libraries were diluted to 20 pM using hybridization buffer purchased from Illumina. Cluster amplification of denatured templates was performed according to the manufacturer’s protocol (Illumina). Flowcells were sequenced using HiSeq 2500 v3 Sequencing-by-Synthesis Kits (2 × 100 bp reads) and then analyzed using RTA v.1.12.4.2 or later.

### Sequence data processing

Using BWA v0.7.5a^35^, all of the raw data without merging of overlapping reads were aligned to the hg19 human reference creating BAM files. SAMtools v0.1.18^36^, GATK v2.2-25^37^, and Picard v1.93 were used for sorting SAM/BAM files, local realignment, and duplicate markings, respectively. Through the process, we filtered reads to remove duplicates, improper pairs, and off-target reads. SNVs and indels were detected by MuTect 1.1.4^38^ and Pindel 0.2.5a4^39^.

### Performance test

The performance of variant detection for base substitutions and Indels was evaluated based on a previously reported strategy^9^, with minor modifications as described below.

#### Base substitution detection performance

To determine the variants present in normal cell lines used for the evaluation of base substitution detection, all 10 HapMap DNA samples from the 1000 Genomes Project^40^ were sequenced individually at high depth (targeting >1000× coverage by non-PCR duplicate read pairs) to detect base substitutions (Supplementary Table S1). Base substitutions consistent with a homozygous (MAF > 90%) or heterozygous (40% ≤ MAF ≤ 60%) state were listed and used in the test set. The 10 normal cell lines were pooled to create test specimens that included 4840 variants across the targeted regions and spanned a broad range of MAF (5–100%). The expected MAF for each test base substitution in the pooled samples was calculated based on the number of alternate alleles present in mix constituents and on mixing ratios.

#### Indel detection performance

For indel validation, genomic DNA samples from 10 tumor cell lines (ATCC) were sequenced individually at high depth (targeting >1000× coverage by non-PCR duplicate read pairs). To make test specimens that had indel variants with the various MAF, all or subsets of 10 cell lines were pooled in three different ways. The three pools included a total of 70 indel variants predicted to have a broad range of MAF (5–20%). The expected MAFs for indels in pooled samples were calculated based on the MAF of that indel in the individual tumor cell line in each of the pool constituents and on mixing ratios.

#### Background error rate estimation

To estimate the background error rate, a pool of genomic DNAs from 10 HapMap cell lines were used to create libraries, except for the cross-contamination estimation described below. High depth sequencing data of individual 10 cell lines were used to determine background alleles in the pool. Among all possible alleles at every position of target regions (total 8,687,544 = 2,171,886 bp × 4 nucleotides), alleles with a frequency ≤1% in all 10 cell lines were listed as background alleles (6,525,673). Frequencies of the background alleles in the test specimens were calculated as the number of reads supporting the background allele divided by the total read count after the removal of duplicates.

#### Cross-contamination

To evaluate cross-contamination caused by multiplexed hybrid selection, libraries individually constructed from eight HapMap cell lines DNA (listed in Supplementary Table S3), a subset of cell lines used in test for base substitution detection, were subjected to solution-phase hybrid capture in either single-plex or eight-plex format. Based on high depth sequencing data of individual cell lines, alleles with a frequency of greater than 10% at every position in the target region were regarded as a genotype of a given sample. For each sample, background alleles with frequencies lower than 1% at every position in target regions were listed and divided into either a ‘test’ group or a ‘control’ group. The control group consisted of background alleles common to all eight samples, whereas the test group was a collection of alleles that were background alleles in a given sample but a genotype in any of the other seven samples. Thus, all eight samples shared the same control group, but each sample had a unique test groups. Assuming minimal cross-contamination between samples, a background allele in a sample would hardly increase the frequency of the allele in the other samples, which was relevant to the control group. In contrast, a genotype allele, typically with 50% or 100% allele frequency, in a sample would influence results significantly if the allele was a background allele in the other samples, which might occur in the test group. After sequencing libraries enriched by either single- or eight-plex hybrid selection, the data size of each sample was down-sampled to a total of 8.6 M reads. Background error rates in the two groups were calculated for each sample and averaged.

## Additional Information

**Accession codes:** Raw sequencing data were deposited in the Sequence Read Archive with accession number SRP070878.

**How to cite this article**: Chung, J. *et al*. The minimal amount of starting DNA for Agilent’s hybrid capture-based targeted massively parallel sequencing. *Sci. Rep.* **6**, 26732; doi: 10.1038/srep26732 (2016).

## Supplementary Material

Supplementary Information
