# Supplementary material for: The minimal amount of starting DNA for Agilent’s hybrid capture-based targeted massively parallel sequencing
Source: Sci Rep. 2016 May 25;6:26732. doi: 10.1038/srep26732 (PMC4879621; doi:10.1038/srep26732)
Supplement: Supplementary Information [file srep26732-s1.pdf]

The minimal amount of starting DNA for Agilent's hybrid capture-based targeted massively parallel sequencing

Jongsuk Chung[1,2,3#], Dae-Soon Son[1,2#], Hyo-Jeong Jeon[2#], Kyoung-Mee Kim[4], Gahee Park[2], Gyu Ha Ryu[5], Woong-Yang Park[2,3*], Donghyun Park[1,2*]

[1] Samsung Biomedical Research Institute, Samsung Advanced Institute of Technology, Samsung Electronics Co. Ltd., Seoul 06351, Korea

[2] Samsung Genome Institute, Samsung Medical Center, Seoul 06351, Korea

[3]Department of Molecular Cell Biology, Sungkyunkwan University School of Medicine, Suwon 16419, Korea

[4]Department of Pathology and Translational Genomics, Sungkyunkwan University School of Medicine, Suwon 16419, Korea

[5]Office of Research and Development Strategy & Planning, Samsung Medical Center, Seoul 06351, Korea

[#] These authors contributed equally to this work.

* To whom correspondence should be addressed

Woong-Yang Park
E-mail: woongyang.park@samsung.com
Tel: +82 2 3410 6128
Fax: +82 2 2148 9819

Donghyun Park
E-mail: dh0307.park@samsung.com
Tel: +82 2 3410 2954
Fax: +82 2 2148 9819

SUPPLEMENTARY INFORMATION

Supplementary Figures S1–5

Supplementary Tables S1–7

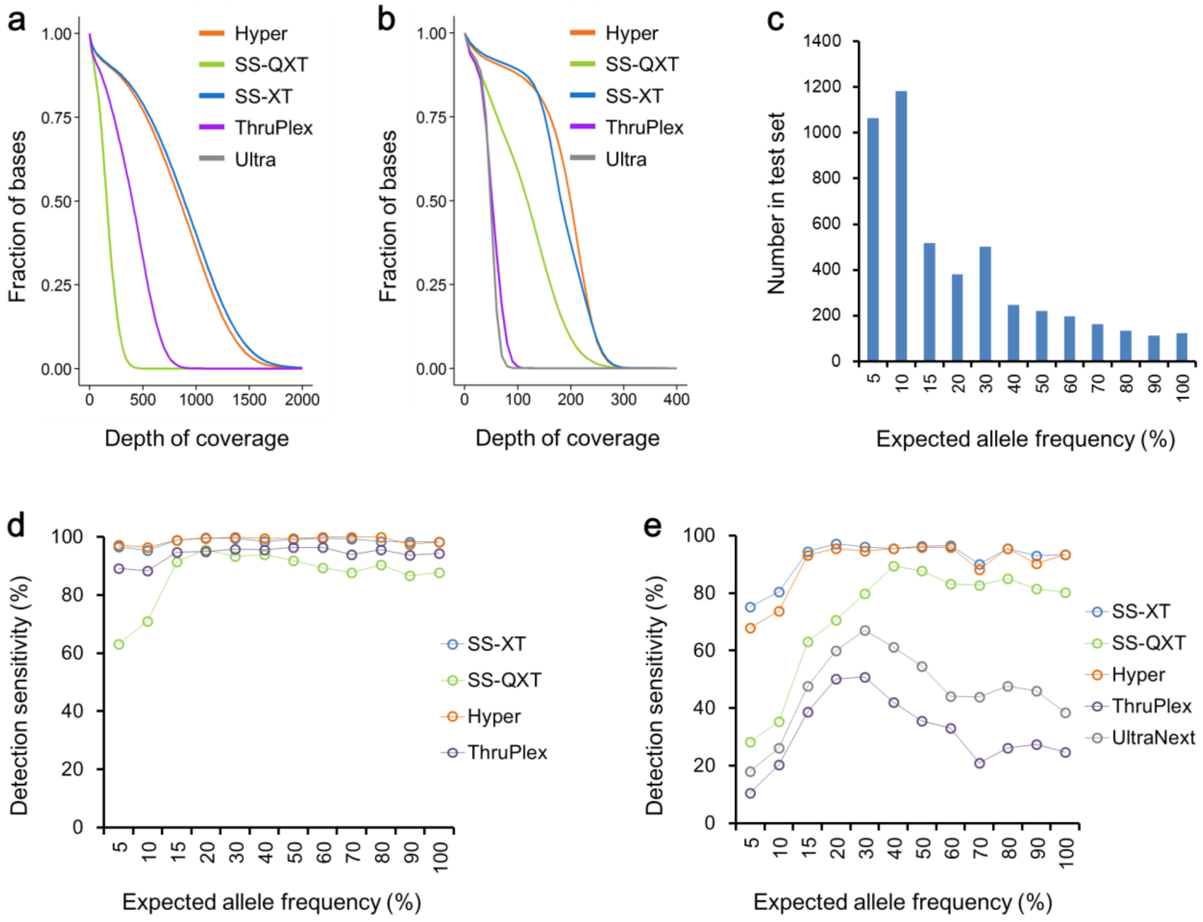

Supplementary Figure S1 Comparison of kits using HapMap cell line DNA. (**a, b**) Coverage efficiency comparison. Libraries were constructed using 200 ng (**a**) or 10 ng (**b**) of input DNA. Coverage efficiency was visualized as the percent of the total targeted bases covered at particular depths. Data was averaged from two sets of libraries from two HapMap cell-line pools. (**c**) Expected allele frequencies of base substitution alterations within the test set, the pool of 10 HapMap cell lines. (**d, e**) Base substitution detection sensitivity of libraries using 200 ng (**d**) or 10 ng (**e**) of input DNA was plotted against expected allele frequencies of base substitution alterations (x-axis).

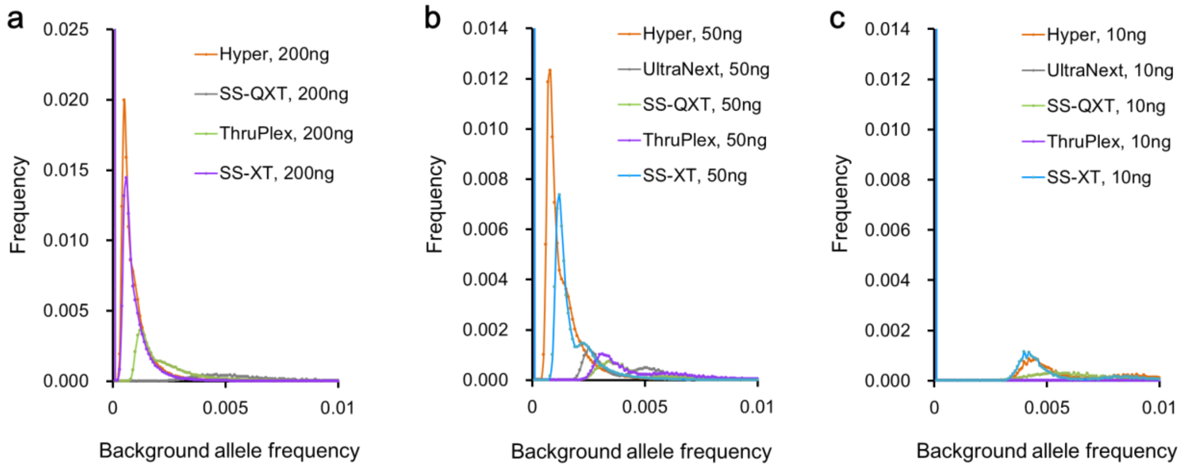

Supplementary Figure S2 Analysis of background error rate. Libraries were constructed by using 200 ng (**a**), 50 ng (**b**), and 10 ng (**c**) of input DNA from the pool of 10 HapMap cell lines. The x-axis denotes the frequency of background alleles, and the y-axis denotes the fraction of alleles with the background rate designated on the x-axis. Details are given in Materials and Methods.

a

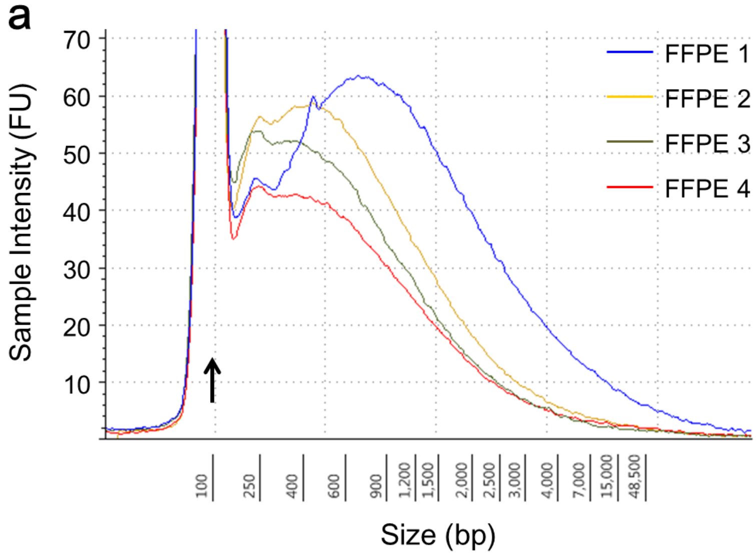

b

	FFPE 1	FFPE 2	FFPE 3	FFPE 4
Median size (bp)	695	439	247	262
qPCR ΔCq	0.8	2.0	2.8	4.3

Supplementary Figure S3 Quality estimation of DNAs from four FFPE samples. (**a**) The distribution of fragment size was measured by Agilent 2200 TapeStation. (**b**) Median fragment sizes based on fragment size distribution and qPCR results from Illumina FFPE QC assay were summarized. The manufacturer recommended that samples with ΔCq values below or equal to 2 were adequate for subsequent processes.

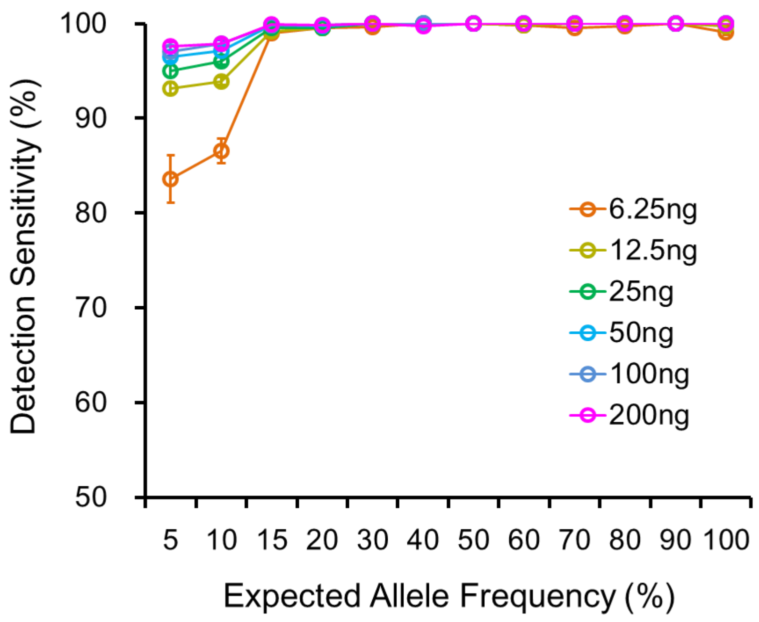

Supplementary Figure S4 Base substitution detection sensitivity after removal of regions with low coverage bias. Base substitution detection sensitivity of libraries was plotted against expected allele frequencies of base substitution alterations (x-axis). Target regions showing below 200× mean coverage calculated from 22 samples were removed. Values are expressed as mean ± s.e.m. (n = 3 or 4).

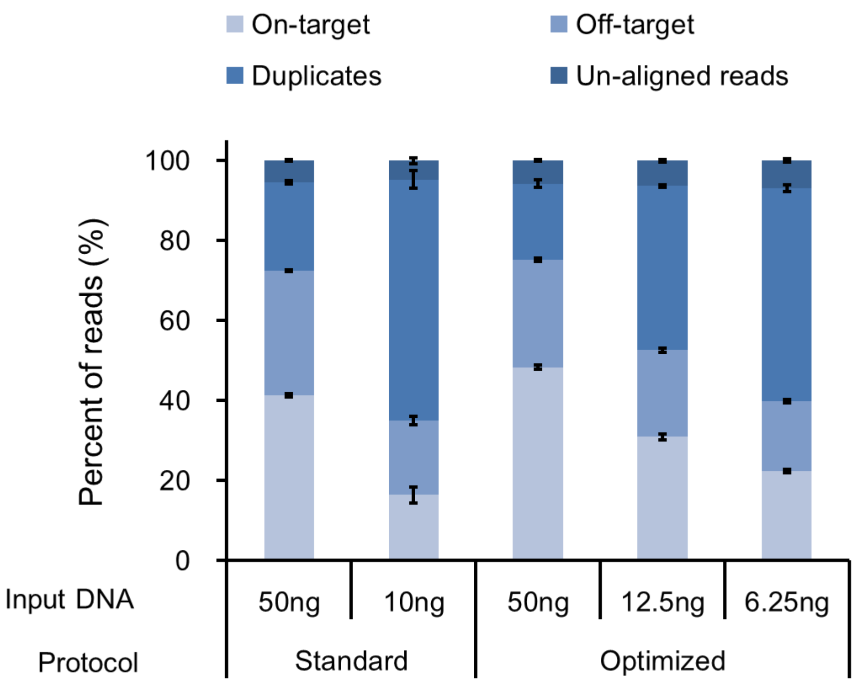

Supplementary Figure S5 Comparison of sequencing metrics between the standard SureSelect-XT protocol and the optimized protocol. All sequencing data were down-sample from each raw fastq data to to a 1233× mean depth of coverage.

Sample ID	Normal HapMap cell line	Input amount (ng)	Library prep. platform	Total reads	Uniquely aligned reads	Fraction of uniquely aligned reads (%)	On target bases	Mean target cover age	Fraction target covered (> X100)
NA07014	NA07014	200	sureselect XT	48,352,684	46,562,322	96.30	2,487,356,303	1304	0.99
NA10840	NA10840	200	sureselect XT	49,638,362	47,819,422	96.34	2,574,029,359	1345	0.99
NA18595	NA18595	200	sureselect XT	50,405,332	48,594,669	96.41	2,579,061,025	1352	0.99
NA18957	NA18957	200	sureselect XT	47,576,932	45,731,451	96.12	2,427,594,243	1266	0.99
NA18488	NA18488	200	sureselect XT	59,276,564	56,157,007	94.74	2,801,088,033	1463	0.99
NA18511	NA18511	200	sureselect XT	48,107,532	46,153,978	95.94	2,434,581,315	1267	0.99
NA18867	NA18867	200	sureselect XT	48,849,822	46,720,701	95.64	2,409,818,907	1257	0.99
NA18924	NA18924	200	sureselect XT	50,522,710	48,086,917	95.18	2,505,624,571	1305	0.99
NA19108	NA19108	200	sureselect XT	49,303,908	47,071,648	95.47	2,437,246,533	1277	0.99
NA19114	NA19114	200	sureselect XT	48,784,804	46,766,020	95.86	2,493,151,449	1312	0.99
XX_compare_sureXT_200	XX group	200	sureselect XT	49,002,072	46,975,306	95.86	2,458,996,331	1297	0.99
XX_compare_sureXT_50	XX group	50	sureselect XT	44,286,772	41,833,230	94.46	1,420,047,016	718	0.99
XX_compare_sureXT_10	XX group	10	sureselect XT	38,227,832	36,704,836	96.02	476,753,261	224	0.98
XX_compare_sureQXT_200	XX group	200	sureselect QXT	48,262,080	45,909,443	95.13	419,998,162	172	0.87
XX_compare_sureQXT_50	XX group	50	sureselect QXT	43,926,316	42,626,442	97.04	511,957,352	249	0.96
XX_compare_sureQXT_10	XX group	10	sureselect QXT	44,591,342	42,911,450	96.23	342,645,331	151	0.81
XX_compare_KAPA_200	XX group	200	KAPA Hyper	64,202,830	61,524,079	95.83	2,819,117,477	1481	0.99
XX_compare_KAPA_50	XX group	50	KAPA Hyper	55,616,144	53,384,253	95.99	2,014,218,567	1038	0.99
XX_compare_KAPA_10	XX group	10	KAPA Hyper	48,894,826	46,684,325	95.48	434,425,487	209	0.97
XX_compare_Thru_200	XX group	200	Thruplex	53,715,794	48,589,658	90.46	969,042,998	586	0.97
XX_compare_Thru_50	XX group	50	Thruplex	45,638,922	42,284,439	92.65	438,794,085	262	0.95
XX_compare_Thru_10	XX group	10	Thruplex	51,282,602	47,207,106	92.05	77,440,153	45	0.00
XX_compare_NEB_50	XX group	50	NEB ultra	59,350,050	54,345,772	91.57	579,342,078	348	0.97
XX_compare_NEB_10	XX group	10	NEB ultra	60,346,910	53,685,808	88.96	90,377,513	52	0.02
YY_compare_sureXT_200	YY group	200	sureselect XT	45,664,936	43,538,633	95.34	2,256,988,729	1178	0.99
YY_compare_sureXT_50	YY group	50	sureselect XT	48,510,256	46,010,256	94.85	1,535,279,299	763	0.99
YY_compare_sureXT_10	YY group	10	sureselect XT	39,562,812	37,405,592	94.55	366,113,421	167	0.98
YY_compare_sureQXT_200	YY group	200	sureselect QXT	42,464,558	40,919,704	96.36	587,419,980	254	0.95
YY_compare_sureQXT_50	YY group	50	sureselect QXT	48,824,386	46,339,998	94.91	451,898,878	183	0.89
YY_compare_sureQXT_10	YY group	10	sureselect QXT	46,364,380	44,922,068	96.89	359,395,443	177	0.89
YY_compare_KAPA_200	YY group	200	KAPA Hyper	56,771,114	54,528,806	96.05	2,555,966,895	1353	0.99
YY_compare_KAPA_50	YY group	50	KAPA Hyper	49,765,362	47,679,828	95.81	1,899,327,651	999	0.99
YY_compare_KAPA_10	YY group	10	KAPA Hyper	43,048,570	40,664,108	94.46	441,623,694	211	0.97
YY_compare_Thru_200	YY group	200	Thruplex	58,175,184	52,152,482	89.65	901,079,165	543	0.99
YY_compare_Thru_50	YY group	50	Thruplex	49,211,698	45,494,753	92.45	368,859,689	218	0.99
YY_compare_Thru_10	YY group	10	Thruplex	48,364,886	44,775,966	92.58	117,079,456	68	0.00
YY_compare_NEB_50	YY group	50	NEB ultra	59,669,474	55,202,407	92.51	653,386,008	394	0.94

Supplementary Table S1

Summary of sequencing metrics for kit comparison using genomic DNAs from HapMap normal cell-lines.

Normal HapMap cell line	Number of homozygous base substitutions	Number of heterozygous base substitution	Sum
NA07014	532	884	1416
NA10840	517	939	1456
NA18595	456	872	1328
NA18957	556	868	1424
NA18488	674	1153	1827
NA18511	575	1273	1848
NA18867	603	1199	1802
NA18924	613	997	1610
NA19108	563	1033	1596
NA19114	561	1280	1841
Total			4840

Supplementary Table S2

Summary of cell-lines used for evaluation of base substitution detection.

Sample ID	FFPE ID	Input amount (ng)	Library prep. platform	Total reads	Uniquely aligned reads	Fraction of uniquely aligned reads (%)	On target bases	Mean target coverage	Fraction target covered (> X100)
XX_XT_300_1	FFPE1	300	sureselect XT	53,719,180	45,732,754	85.13	1,314,625,370	1053	0.99
XX_XT_300_2	FFPE2	300	sureselect XT	54,094,486	50,208,104	92.82	1,406,379,307	1127	0.99
XX_XT_300_3	FFPE3	300	sureselect XT	53,696,836	45,276,926	84.32	1,065,405,986	853	0.99
XX_XT_300_4	FFPE4	300	sureselect XT	50,676,414	43,701,920	86.24	535,946,951	429	0.98
XX_XT_100_1	FFPE1	100	sureselect XT	56,940,584	49,053,657	86.15	1,172,374,138	939	0.99
XX_XT_100_2	FFPE2	100	sureselect XT	60,892,264	53,844,471	88.43	1,047,794,555	839	0.99
XX_XT_100_3	FFPE3	100	sureselect XT	51,570,722	44,729,758	86.73	644,522,338	516	0.99
XX_XT_100_4	FFPE4	100	sureselect XT	50,139,156	44,492,070	88.74	250,794,810	201	0.90
XX_XT_50_1	FFPE1	50	sureselect XT	60,086,136	56,034,567	93.26	786,766,976	631	0.88
XX_XT_50_2	FFPE2	50	sureselect XT	50,089,236	44,919,034	89.68	637,094,670	510	0.97
XX_XT_50_3	FFPE3	50	sureselect XT	47,795,532	42,097,401	88.08	300,603,254	241	0.83
XX_XT_50_4	FFPE4	50	sureselect XT	39,348,630	35,169,686	89.38	107,769,760	86	0.21
XX_HYPER_300_1	FFPE1	300	KAPA Hyper	50,420,226	45,980,193	91.19	1,389,027,755	1113	0.99
XX_HYPER_300_2	FFPE2	300	KAPA Hyper	48,392,400	44,713,529	92.40	1,286,292,381	1030	0.99
XX_HYPER_300_3	FFPE3	300	KAPA Hyper	57,445,352	53,235,162	92.67	1,182,335,950	947	0.99
XX_HYPER_300_4	FFPE4	300	KAPA Hyper	49,339,714	46,512,500	94.27	696,742,899	558	0.99
XX_HYPER_100_1	FFPE1	100	KAPA Hyper	46,799,174	43,893,933	93.79	993,931,395	796	0.98
XX_HYPER_100_2	FFPE2	100	KAPA Hyper	47,768,526	45,242,763	94.71	893,368,521	716	0.99
XX_HYPER_100_3	FFPE3	100	KAPA Hyper	51,530,230	49,177,954	95.44	574,702,007	461	0.99
XX_HYPER_100_4	FFPE4	100	KAPA Hyper	48,791,226	46,554,043	95.41	291,200,353	233	0.95
XX_HYPER_50_1	FFPE1	50	KAPA Hyper	20,719,948	19,620,172	94.69	388,650,101	312	0.63
XX_HYPER_50_2	FFPE2	50	KAPA Hyper	51,156,084	48,387,325	94.59	589,792,088	473	0.96
XX_HYPER_50_3	FFPE3	50	KAPA Hyper	47,301,696	44,038,004	93.10	352,063,267	282	0.96
XX_HYPER_50_4	FFPE4	50	KAPA Hyper	42,620,024	40,376,488	94.74	142,837,029	115	0.44

Supplementary Table S3

Summary of sequencing metrics for kit comparison using genomic DNAs from FFPE samples.

Normal HapMap cell line	Number of background alleles in the control group	Number of background alleles in the test group
NA07014	1,404,712	694
NA10840	1,404,712	718
NA18488	1,404,712	619
NA18511	1,404,712	665
NA18595	1,404,712	733
NA18867	1,404,712	628
NA18924	1,404,712	661
NA18957	1,404,712	693
Total	1,404,712	5,411

Supplementary Table S4

Summary of cell lines used for the evaluation of cross-contamination.

Sample ID	Batch	Input amount (ng)	Total reads	Uniquely aligned reads	Fraction of uniquely aligned reads (%)	On target bases	Mean target coverage	Fraction target covered (> X100)
XX_validate_625_1_1	1	6.25	25,503,752	23,529,657	92.26	329,384,796	264	0.97
XX_validate_625_2_1	1	6.25	32,757,036	30,962,059	94.52	381,880,516	306	0.98
XX_validate_125_1_1	1	12.5	46,090,736	42,658,526	92.55	583,201,362	467	0.99
XX_validate_125_2_1	1	12.5	51,336,910	48,444,352	94.37	695,823,793	558	0.99
XX_validate_25_1_1	1	25	62,332,428	58,278,619	93.5	1,070,930,451	858	0.99
XX_validate_50_1_1	1	50	49,386,328	46,352,319	93.86	1,224,726,315	981	0.99
XX_validate_100_1_1	1	100	57,965,906	54,372,852	93.8	1,638,722,430	1314	0.99
XX_validate_200_1_1	1	200	63,301,576	58,771,851	92.84	1,895,208,844	1518	0.99
XX_validate_625_1_2	2	6.25	38,207,212	35,892,598	93.94	375,596,047	301	0.98
XX_validate_125_1_2	2	12.5	35,698,540	33,334,593	93.38	578,129,664	463	0.98
XX_validate_25_1_2	2	25	50,695,312	47,590,815	93.88	970,966,852	778	0.99
XX_validate_50_1_2	2	50	48,845,446	45,950,152	94.07	1,248,398,183	1001	0.99
XX_validate_100_1_2	2	10	52,410,880	49,527,535	94.5	1,512,821,555	1212	0.99
XX_validate_200_1_2	2	200	60,824,058	57,528,645	94.58	1,883,373,314	1509	0.99
XX_validate_125_1_3	3	12.5	54,959,488	51,920,625	94.47	806,989,300	489	0.99
XX_validate_25_1_3	3	25	57,041,342	54,222,622	95.06	1,272,918,856	777	0.99
XX_validate_50_1_3	3	50	46,392,896	43,828,500	94.47	1,472,671,766	906	0.99
XX_validate_100_1_3	3	10	64,769,486	61,465,926	94.9	2,175,834,535	1334	0.99
XX_validate_200_1_3	3	200	70,602,794	66,983,608	94.87	1,957,733,515	1569	0.99
XX_validate_625_1_4	4	6.25	29,460,350	27,357,049	92.86	336,804,027	270	0.95
XX_validate_125_1_4	4	12.5	44,985,596	42,030,261	93.43	794,283,457	482	0.98
XX_validate_50_1_4	4	50	61,214,202	57,873,418	94.54	1,928,443,680	1186	0.99
XX_validate_100_1_4	4	10	58,125,254	54,464,376	93.7	2,015,663,221	1244	0.99
XX_validate_200_1_4	4	200	68,888,004	65,222,669	94.68	2,579,360,965	1589	0.99

Supplementary Table S5

Summary of sequencing metrics for the performance evaluation for SNV detection sensitivity using the optimized protocol.

Tumor cell line	Gene	Indel type	Indel length (bp)	Measured mutant allele frequency	Genomic coordinates (hg19)	Reference sequence	Alternate sequence
A549	*SMO*	non-frameshift insertion	3	0.2381	chr7:128829039	-	GCT
CCRF-CEM	*HNF1A*	frameshift deletion	1	0.1134	chr12:121432115	G	-
	NOTCH1	non-frameshift insertion	36	0.2468	chr9:139399362	-	GCAGGAAGTGGAAGGAGC TGTTGTGGGGAAGGCGCG
HCT-15	*APC*	frameshift deletion	1	0.447	chr5:112175539	C	-
	BRCA2	frameshift deletion	2	0.31	chr13:32912090	TG	-
NCI-H128	*ARID1A*	frameshift deletion	1	0.285	chr1:27092811	G	-
	RB1	frameshift deletion	1	0.4776	chr13:48951087	A	-
NCI-H727	*IDH2*	frameshift substitution	1	0.3122	chr15:90631943	CCAT	CAA
	TP53	non-frameshift insertion	9	0.1216	chr17:7578434	-	ACTGCTTGT
SW48	*ARID1A*	frameshift deletion	1	0.3347	chr1:27023904	G	-
	ARID1A	frameshift deletion	1	0.3327	chr1:27101402	C	-
	ARID1B	non-frameshift deletion	3	0.25	chr6:157099982	GGC	-
	ATRX	frameshift deletion	2	0.3231	chrX:76938089	TC	-
	BRCA2	frameshift deletion	1	0.3051	chr13:32913559	A	-
	BRCA2	frameshift deletion	1	0.3104	chr13:32913837	A	-
	EZH2	frameshift deletion	1	0.2217	chr7:148515025	C	-
	FBXW7	frameshift deletion	1	0.2975	chr4:153244156	C	-
	FGFR3	frameshift deletion	1	0.2857	chr4:1801138	G	-
	NTRK1	frameshift deletion	1	0.3264	chr1:156846308	C	-
	PTCH1	frameshift deletion	1	0.3193	chr9:98211549	G	-
	SMO	frameshift deletion	1	0.3812	chr7:128852004	C	-
TUR	*PTEN*	frameshift insertion	4	0.9388	chr10:89692903	-	CGCC
RL95-2	*ARID1A*	frameshift deletion	1	0.3091	chr1:27087373	C	-
	ARID1B	frameshift deletion	1	0.3051	chr6:157405954	G	-
	ARID1B	non-frameshift insertion	3	0.4407	chr6:157100396	-	CGC
	ATM	frameshift deletion	1	0.3036	chr11:108172510	A	-
	BRAF	frameshift deletion	1	0.3041	chr7:140482927	G	-
	BRCA2	frameshift insertion	1	0.3031	chr13:32913952	-	A
	BRCA2	frameshift insertion	1	0.3119	chr13:32912770	-	T
	JAK1	frameshift deletion	1	0.3001	chr1:65305357	T	-
	JAK1	frameshift deletion	1	0.3115	chr1:65306997	T	-
	NTRK1	frameshift insertion	1	0.2966	chr1:156848967	-	C
	PTEN	frameshift insertion	1	0.3122	chr10:89720811	-	A
	PTEN	frameshift deletion	1	0.3102	chr10:89720812	A	-
	TP53	non-frameshift deletion	3	0.4602	chr17:7578195	CAC	-

Supplementary Table S6a

Summary of tumor cell lines and indel variants used for the evaluation of indel detection. NCI-H146 and THP-1 were not listed in the table because they did not have any indel variants verified in sequence data.

Pool of ten cell lines: pool A

A549	CCRF-CEM	HCT-15	NCI-H128	NCI-H146
NCI-H727	SW48	THP-1	TUR	RL95-2

Pools of five cell lines (1): pool B

A549	CCRF-CEM	HCT-15	NCI-H128	NCI-H146

Pools of five cell lines (2): pool C

NCI-H727	SW48	THP-1	TUR	RL95-2

Supplementary Table S6b

Summary of tumor cell line pools used for the evaluation of indel detection

Sample ID	Input amount (ng)	Total reads	Uniquely aligned reads	Fraction of uniquely aligned reads (%)	On target bases	Mean target coverage	Fraction target covered (> X100)
InDel_group_A_200	200	48,970,062	46,596,599	95.15	1,643,589,721	1318	0.99
InDel_group_B_200	200	43,054,566	41,096,524	95.45	1,446,541,437	1160	0.99
InDel_group_C_200	200	51,010,360	48,228,285	94.55	1,688,905,376	1353	0.99
InDel_group_A_100	100	40,752,556	38,724,456	95.02	1,317,464,670	1056	0.99
InDel_group_B_100	100	57,715,312	54,372,947	94.21	1,694,971,205	1358	0.99
InDel_group_C_100	100	44,248,304	41,946,152	94.8	1,404,055,162	1125	0.99
InDel_group_A_50	50	54,723,632	51,664,046	94.41	1,363,927,746	1093	0.99
InDel_group_B_50	50	51,858,072	48,723,648	93.96	1,308,315,347	1049	0.99
InDel_group_C_50	50	60,249,092	56,616,241	93.97	1,484,658,608	1190	0.99
InDel_group_A_25	25	54,771,886	51,426,025	93.89	1,124,183,380	901	0.99
InDel_group_B_25	25	57,665,264	53,523,905	92.82	1,073,595,129	860	0.99
InDel_group_C_25	25	54,981,408	51,667,676	93.97	1,103,008,974	884	0.99

Supplementary Table S7

Summary of sequencing metrics for the performance evaluation for indel detection sensitivity using the optimized protocol.
